# Genetic ablation of ataxin-2 increases several global translation factors in their transcript abundance but decreases translation rate

**DOI:** 10.1007/s10048-015-0441-5

**Published:** 2015-02-27

**Authors:** M. Fittschen, I. Lastres-Becker, M. V. Halbach, E. Damrath, S. Gispert, M. Azizov, M. Walter, S. Müller, G. Auburger

**Affiliations:** 1 Experimental Neurology, Goethe University Medical School, Theodor Stern Kai 7, 60590 Frankfurt am Main, Germany; 2 Institute for Medical Genetics, Eberhard-Karls-University, 72076 Tübingen, Germany; 3 Molecular BioSciences, Biocenter, Goethe University, 60590 Frankfurt am Main, Germany; 4 Present Address: Centro de Investigación Biomédica en Red sobre Enfermedades Neurodegenerativas (CIBERNED), Instituto de Investigación Sanitaria La Paz (IdiPAZ), Departamento de Bioquímica e Instituto de Investigaciones Biomédicas “Alberto Sols” CSIC-UAM, Facultad de Medicina, Universidad Autónoma de Madrid, Madrid, Spain

**Keywords:** Spinocerebellar ataxia, Amyotrophic lateral sclerosis, ATXN2, RNA processing, Ribosomal translation, Ribosomal S6 phosphorylation, Global protein synthesis rates, Cellular stress

## Abstract

Spinocerebellar ataxia type 2 (SCA2) and amyotrophic lateral sclerosis (ALS) are neurodegenerative disorders, caused or modified by an unstable CAG-repeat expansion in the SCA2 gene, which encodes a polyglutamine (polyQ) domain expansion in ataxin-2 (ATXN2). ATXN2 is an RNA-binding protein and interacts with the poly(A)-binding protein PABPC1, localizing to ribosomes at the rough endoplasmic reticulum. Under cell stress, ATXN2, PABPC1 and small ribosomal subunits are relocated to stress granules, where mRNAs are protected from translation and from degradation. It is unknown whether ATXN2 associates preferentially with specific mRNAs or how it modulates RNA processing. Here, we investigated the RNA profile of the liver and cerebellum from *Atxn2* knockout (*Atxn2* ^−/−^) mice at two adult ages, employing oligonucleotide microarrays. Prominent increases were observed for *Lsm12*/*Paip1* (>2-fold), translation modulators known as protein interactor/competitor of ATXN2 and for *Plin3*/*Mttp* (>1.3-fold), known as apolipoprotein modulators in agreement with the hepatosteatosis phenotype of the *Atxn2* ^−/−^ mice. Consistent modest upregulations were also observed for many factors in the ribosome and the translation/secretion apparatus. Quantitative reverse transcriptase PCR in liver tissue validated >1.2-fold upregulations for the ribosomal biogenesis modulator *Nop10*, the ribosomal components *Rps10*, *Rps18*, *Rpl14*, *Rpl18*, *Gnb2l1*, the translation initiation factors *Eif2s2*, *Eif3s6*, *Eif4b*, *Pabpc1* and the rER translocase factors *Srp14*, *Ssr1*, *Sec61b*. Quantitative immunoblots substantiated the increased abundance of NOP10, RPS3, RPS6, RPS10, RPS18, GNB2L1 in SDS protein fractions, and of PABPC1. In mouse embryonal fibroblasts, ATXN2 absence also enhanced phosphorylation of the ribosomal protein S6 during growth stimulation, while impairing the rate of overall protein synthesis rates, suggesting a block between the enhanced translation drive and the impaired execution. Thus, the physiological role of ATXN2 subtly modifies the abundance of cellular translation factors as well as global translation.

**Electronic supplementary material:**

The online version of this article (doi:10.1007/s10048-015-0441-5) contains supplementary material, which is available to authorized users.

## Introduction

Spinocerebellar ataxia type 2 (SCA2) is an autosomal dominantly inherited neurodegenerative disorder caused by large expansions in an unstable polyglutamine domain within the protein ataxin-2 (ATXN2), probably through a toxic gain-of-function mechanism [1–3]. Intermediate size polyglutamine expansions within ATXN2 were more recently implicated as risk factor of the motor neuron disease amyotrophic lateral sclerosis (ALS), of the basal ganglia tauopathy progressive supranuclear palsy (PSP) and of the preferential midbrain degeneration in Parkinson’s disease (PD) [4–7]. A recent survey of modifier genes in *Saccharomyces cerevisiae*, *Caenorhabditis elegans* and *Drosophila melanogaster* concluded that ataxin-2 orthologues are generic modifiers that affect multiple if not all neurodegenerative diseases [8]. Furthermore, the deletion of *Atxn2* in mouse as well as single nucleotide polymorphisms at the chromosomal *ATXN2* locus in human implicated ATXN2 in hepatosteatosis, obesity, dyslipidemia, insulin resistance, diabetes mellitus and arterial hypertension [9–17]. Thus, ataxin-2 appears to regulate basic metabolic features, and its chronic loss or accumulation/aggregation result in age-associated diseases.

ATXN2 was described as a cytoplasmic protein with allelic splicing and an age-dependent expression increase, containing 1312 amino acid residues and migrating at a molecular mass of about 150 kDa [18–20]. However, recently, its translation was reported to depend on a methionine start codon 160 amino acids further down, only 5 amino acids upstream from the polyglutamine domain [21]. Its expression was observed mainly in the brain in specific neuronal populations, for example in cerebellar and cerebral cortex, but also in several non-neuronal tissues such as the skeletal muscle, kidney, prostate, thyroid gland and liver [19]. A role of ATXN2 in trophic signalling through direct interaction with the receptor-mediated endocytosis machinery was discovered through protein interaction studies [22–25]. Its subcellular localization to Golgi organelles was claimed initially on the basis of recombinant overexpression. Later studies of endogenous ATXN2 found it to be associated with polysomes and localized it mainly at the rough endoplasmic reticulum (rER). It was rarely observed at the plasma membrane or shuttling to the nucleus in some cells [26–31].

Protein interaction studies found this co-localization of ATXN2 with ribosomes to depend both on the globular Lsm/Lsm-AD domains of ATXN2 that are thought to mediate RNA processing, and on the PAM2 motif of ATXN2 that is known to mediate direct protein interaction with poly(A)-binding protein PABPC1 [29, 32]. PABPC1 acts during translation, simultaneously binding the 3′-poly(A) tail of mRNAs and the 5′-associated translation initiation factor eIF4G, thus promoting mRNA circularisation as an essential step in translation initiation [29, 33–35]. Interestingly, opposing roles for the two interactor proteins were found in the yeast *S. cerevisiae*, where the deletion of the ATXN2 orthologue Pbp1 suppresses the lethality associated with a PAB1 deletion [36]. Opposing roles were also found in *D. melanogaster* fly models of ataxin-3-induced neurodegeneration, where ATXN2 overexpression potentiates the phenotype, while a phenotype rescue was observed after PABPC1 overexpression [37]. To date, it remains unclear how ATXN2 modulates the PABPC1 function.

Lsm domains as those within ATXN2 are highly conserved in proteins that are involved in important processes of RNA metabolism such as RNA modification, splicing and degradation [29, 34, 38]. The putative role of ATXN2 for RNA processing is further substantiated by its interaction with at least five RNA-binding proteins: (i) ataxin-2 binding protein 1 (A2BP1 or RBFOX1) binds to the C-terminus of ATXN2 and has an RNA recognition motif that is highly conserved among RNA-binding proteins [39]. (ii) ATXN2 was found to associate with DEAD/H-box RNA helicase DDX6, a component of stress granules and P-bodies, thus influencing the storage of mRNA in periods of stalled translation as well as the degradation of mRNAs [30]. (iii) The TDP-43 protein with its two RRM (RNA recognition motifs) shows genetic interaction with ATXN2 and associates with the ATXN2 protein in a complex that depends on RNA binding [4]. (iv) The RRM-containing FUS protein interacts with ATXN2 both at the protein level as well as genetically [40]. (v) The FMRP protein is associated with polysomes as a translational repressor and was recently observed to interact with ATXN2 in long-term neuronal habituation processes [41].

Beyond this indirect evidence for a role of ATXN2 in RNA processing, a direct interaction with RNA could recently be demonstrated experimentally [42]. At least in the case of the *PERIOD* clock gene mRNA, ATXN2 activates translation through enhanced association between PABP and the circadian factor TYF, thus signalling “subjective night” and sustaining the circadian clock [43, 44]. Interestingly, ATXN2 was also implicated in microRNA processing during the habituation of olfactory synapses [41, 45].

Yeast evidence elucidated its function for RNAs further. The ATXN2 yeast orthologue Pbp1 was reported to protect the full length of the poly(A) tail of mRNAs and to act as negative regulator of poly(A) nuclease (PAN) activity [36, 46]. Additional studies showed that the deletion of Pbp1 was able to rescue the deleterious growth of double mutants that harboured the deletion of a deadenylase (CCR4 or POP2) together with the deletion of the RNA-binding protein KHD1 [47]. This effect was also achieved by deletion of the ribosomal proteins Rpl12a/b, with Pbp1 being found to interact with these ribosomal subunits [47]. It is still unclear how RNA processing is modulated by the interaction of Pbp1 with Lsm12, a protein with an N-terminal Lsm domain that has been suggested to play a role in mRNA degradation or tRNA splicing [34, 48–50]. Pbp1 overexpression or heat stress induce stress granule formation and sequester the TORC1 kinase complex, thus blunting its signalling that drives cell growth upon nutrient availability [51, 52].

In periods of cell stress, the immediate suppression of protein synthesis is accompanied by formation of transient cytoplasmic foci known as stress granules (SGs), where untranslated mRNAs accumulate together with 40S small ribosomal subunits, PABPC1 and translation initiation factors [53], as well as by enlargement of mRNA degrading P-bodies. In vitro investigations usually trigger SG formation by glucose deprivation or by the administration of oxidative stress via arsenite. ATXN2 was observed to relocalize to SGs together with PABPC1 and associate with P-bodies. Investigation of human cells showed ATXN2 deficiency to prevent SG assembly, while its overexpression reduced the number of P-bodies per cell [30].

To determine whether ATXN2-dependent altered RNA processing modulates steady-state levels of specific mRNAs, the present study used tissues from *Atxn2* knockout (*Atxn2* ^−/−^ or KO) and wild-type (WT) mice, performing unbiased global mRNA profiling. A pattern of consistent upregulations of ribosome/translation-related transcripts was prominent in both approaches. In view of the known association of ATXN2 with the translation initiation factor PABPC1, the validation of these translation-relevant factors and the study of global protein synthesis changes in *Atxn2* ^−/−^ embryonal fibroblasts were prioritized for this manuscript.

## Results

### Screening of the transcriptome by microarrays: liver and cerebellum profile in Atxn2^−/−^ mice until age 6 months

The transcriptome profiles were analysed for 4 conditions with a total of 32 microarray chips. In view of the relevance of ATXN2 for the age-progressive disease hepatosteatosis and SCA2, we used the tissues liver and cerebellum, each collected at ages 6 or 24 weeks (always from 4 *Atxn2* ^−*/*−^ versus 4 *Atxn2* ^*+/+*^ mice). *Atxn2* is known to show strong physiological expression not only in several neuronal populations such as cerebellar Purkinje cells and spinal motor neurons, but also in liver and in the hepatocyte-like tumor cells Hep G2. The absence of *Atxn2* transcripts from the KO tissues was confirmed by the relevant Affymetrix oligonucleotide spots 1419866_s_at, 1460653_at, 1438143_s_at, 1438144_x_at, 1459363_at and 1443516_at. Automated bioinformatic analyses showed 70 additional non-anonymous genes with significant mRNA level upregulation and consistency for all 4 conditions (Suppl. Table 1). Most of the altered factors have roles in RNA translation/processing, ER secretion, lipid metabolism, growth/adhesion or cytoskeletal dynamics. Several observations were in excellent agreement with previous knowledge on ATXN2 biology: (i) More than 6-fold increases were observed by two oligonucleotide spots for *Paip1*, a translation activator that binds to the PAM2-domain of PABPC1 just like ATXN2 [54, 55]; (ii) more than 2-fold increases were documented by two oligonucleotide spots for *Lsm12*, an mRNA degradation or tRNA splicing factor that is known to interact and colocalize with the ATXN2-ortholog Pbp1 in yeast [48, 52]; (iii) a 1.3-fold increase was detected for *Sh3kbp1* (CIN85), an interactor protein of ATXN2 in the receptor endocytosis complex [24]; (iv) a 1.3-fold increase was found also for *Plin3*, a factor important for the biogenesis of lipid droplets [56], which are known to accumulate in *Atxn2* ^−/−^ liver; (v) a 1.4-fold increase was shown by two oligonucleotide spots for *Mttp*, a factor required for the secretion of apolipoproteins and responsible for hepatic steatosis [57]. Modest consistent upregulations were particularly frequent in the RNA translation/processing pathway, with significant >1.2-fold changes for 17 transcripts encoding many proteins of the small and large ribosomal subunit (*Rps2*, *Rps10*, *Rps12*, *Rps15*, *Rps16*, *Rps18*, *Rps26*, *Rpsa*, *Rpl6*, *Rpl8*, *Rpl10*, *Rpl13*, *Rpl14*, *Rpl18*, *Rpl23*, *Rpl29*, *Rpl41*), for the 40S small ribosome subunit component *Gnb2l1* (encoding the RACK1 protein), the ribosomal biogenesis factor *Nop10* (encoding the Nola3 ribonucleoprotein) and the translation initiation factor *Eif2s2*. Other notable changes included a modest increase for the mRNA decay factor *Dcps*, given that ATXN2 modulates the size of P-bodies. Strong >2-fold changes were noted also for the tRNA-editing factor *Deadc1* and for *Drbp1*/*Rbm45*, an RNA-binding factor that aggregates with TDP-43 in ALS neurons [58]. A marked increase was also found for *Ssr1*, a receptor that binds to nascent peptides during translation at the rER to mediate their membrane translocation (on average 1.8-fold). The complete microarray transcriptome results were deposited in the public database GEO (http://www.ncbi.nlm.nih.gov/geo/query/acc.cgi?acc=GSE55177) under the accession number GSE55177. Thus, the main finding from the KO tissues was that *Atxn2* levels correlated inversely with numerous mRNAs that encode factors of RNA processing/translation.

### Candidate analyses by quantitative PCR and immunoblots

**Fig. 1:**
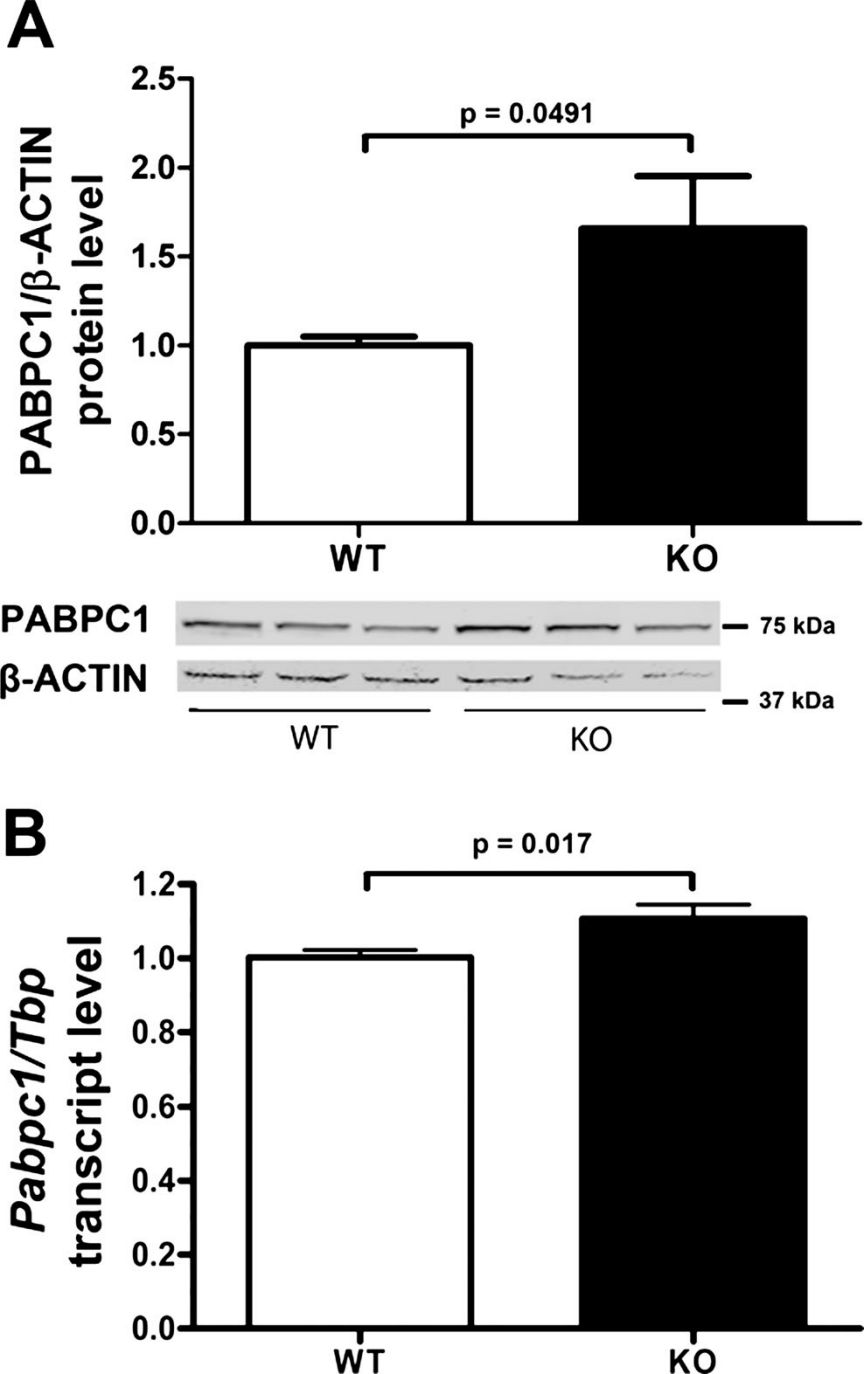
ATXN2 KO upregulates levels of PABPC1 protein and mRNA. **a** A significant upregulation of the PABPC1/beta-ACTIN protein ratio in KO liver at age 6 months (7 *Atxn2* ^−/−^ versus 7 *Atxn2* ^+/+^ tissues) was detected, as shown in a bar graph above and in an immunoblot below. **b** qPCR showed a subtle, but significant upregulation for the ratio *Pabpc1/Tbp* in KO liver at 6 months

For a candidate-driven quantification of effects at the mRNA and protein level and for further validation, we employed the liver from independent animals at age 24 weeks. PABPC1 as a known protein interactor of ATXN2 was first assessed as a positive control. A significant upregulation was demonstrable by quantitative immunoblots (Fig. 1a) as previously published for a cell line with *ATXN2* knockdown [30], and here, through the use of quantitative real-time reverse transcriptase PCR (qPCR), also a subtle significant increase was detected at the mRNA level (Fig. 1b).

**Fig. 2:**
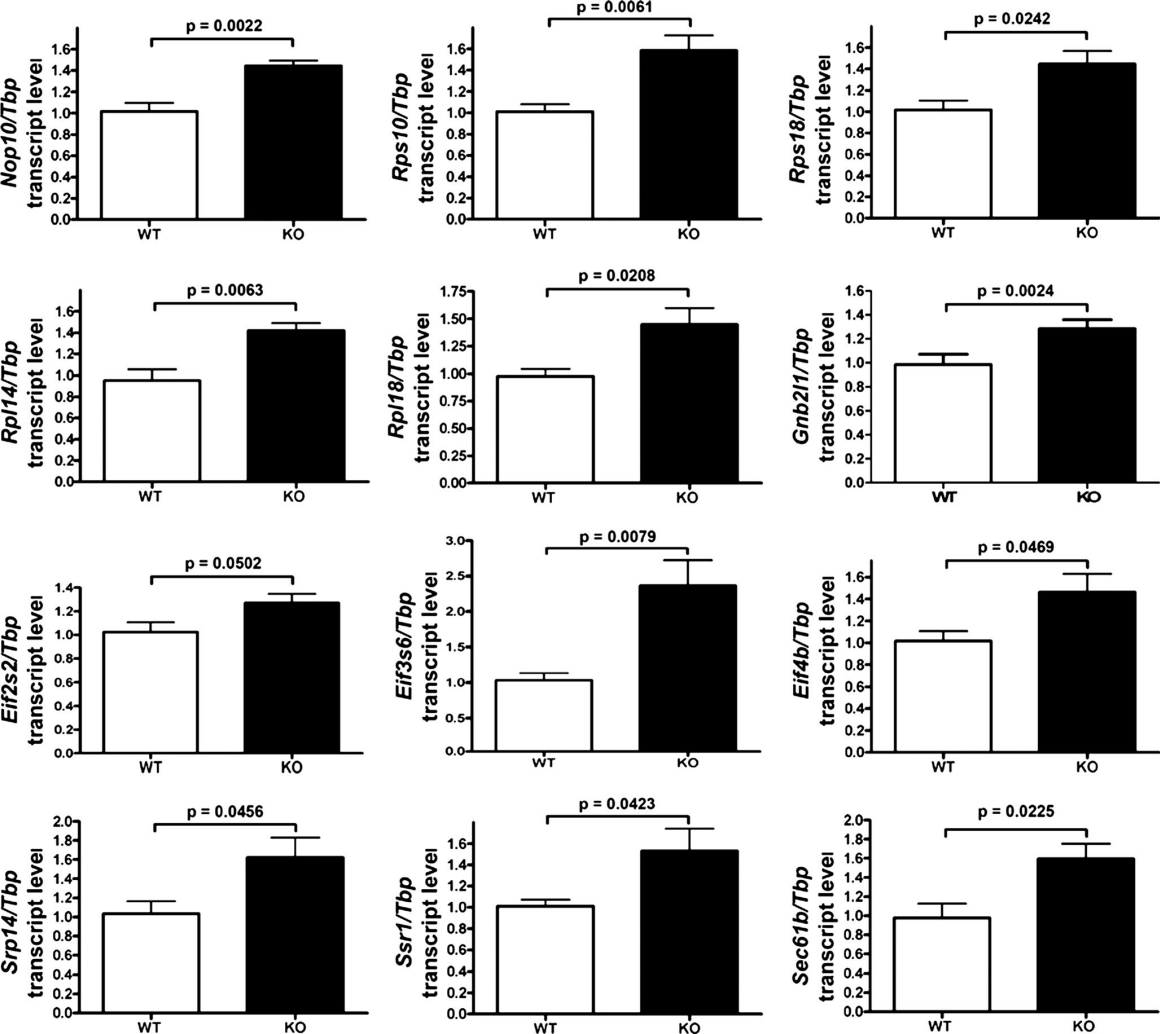
ATXN2 KO upregulates mRNAs of ribosomal/translation factors in qPCR. Modestly but significantly increased transcript levels were consistently found in qPCR of KO liver at age 6 months (12 *Atxn2* ^−/−^ versus 12 *Atxn2* ^+/+^) for twelve factors that represent ribosomal biogenesis (*Nop10*), ribosomal small subunit (*Rps10*, *Rps18*), ribosomal large subunit (*Rpl14*, *Rpl18*), ribosome association with translation factors (*Gnb2l1*), translation initiation complex (*Eif2s2*, *Eif3s6*, *Eif4b*) and rER membrane translocation (*Srp14*, *Ssr1*, *Sec61b*). *Tbp* served as loading control to normalize the levels of the candidate

To distinguish whether ATXN2 has only strong effects on few translation factors, or whether the modest effects in diverse aspects of translation are also valid, we then prioritized 12 factors in the ribosome/translation pathway to represent ribosomal biogenesis (*Nop10*), small and large ribosome subunits (*Rps10* and *Rps18*, *Rpl14* and *Rpl18*), small ribosomal subunit interactors (*Gnb2l1*), translation initiation (*Eif2s2*, *Eif3s6*, *Eif4b*) and rER membrane translocation (*Srp14*, *Ssr1*, *Sec61b*). The latter candidates were also upregulated in the transcriptome surveys but had missed significance after multiple testing corrections in all tissue/age conditions. For all these factors, significant upregulations were consistently reproduced by qPCR (12 *Atxn2* ^*−/−*^ versus 12 *Atxn2* ^*+/+*^), which exceeded the fold-change effect size that had been observed previously for *Pabpc1* (Fig. 2).

**Fig. 3:**
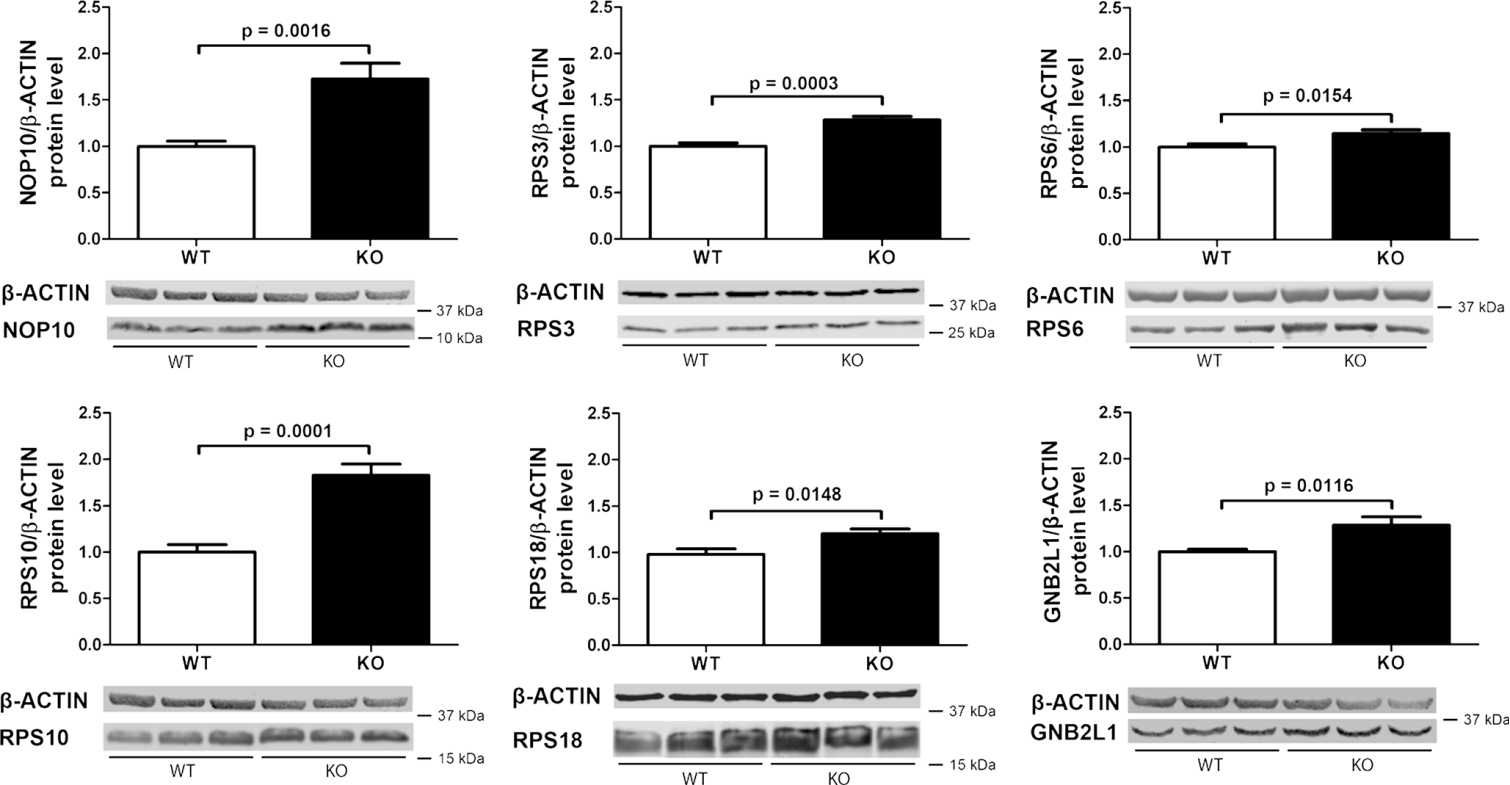
ATXN2 KO upregulates proteins of ribosomal/translation factors. Modestly but significantly enhanced protein levels were consistently found in quantitative immunoblots of RIPA-insoluble, but SDS-soluble extracts from KO liver at age 6 months (7 *Atxn2* ^−/−^ versus 7 *Atxn2* ^+/+^) for six factors that represent ribosomal biogenesis (NOP10/NOLA3), components of the 40S small ribosomal subunit (RPS3, RPS6, RPS10, RPS18), and the association between the 40S small ribosomal subunit and translation initiation factors (GNB2L1/RACK1). Beta-ACTIN served as loading control to normalize the levels of the candidate

Although quantitative immunoblot studies have difficulties to detect changes lower than 2-fold due to non-linear sigmoid membrane binding and enzyme kinetics, we attempted validation at the protein level for candidates where specific antibodies are available. Protein soluble in RIPA buffer (which brings cytosolic factors into solution) did not reveal any significant changes, but proteins solubilized from tissue after SDS treatment (which brings membrane-associated factors into solution) exhibited significant upregulations (in 7 *Atxn2* ^*−/−*^ versus 7 *Atxn2* ^+/+^ tissues) for NOP10, RPS3, RPS6, RPS10, RPS18 and GNB2L1/RACK1 (Fig. 3). These immunoblot observations might suggest that ATXN2 has a stronger influence on ribosomes in association with the membranes of the endoplasmic reticulum than on free ribosomes and polysomes in the cytosol.

Thus, the validation studies corroborate the transcriptome surveys and extend previous reports that ATXN2 interacts with rER ribosome/translation complex elements [26, 29, 30, 41, 43–45, 59], demonstrating ATXN2 to modify their mRNA and protein levels.

### Ataxin-2 deficiency enhances S6 phosphorylation and impairs global protein synthesis

**Fig. 4:**
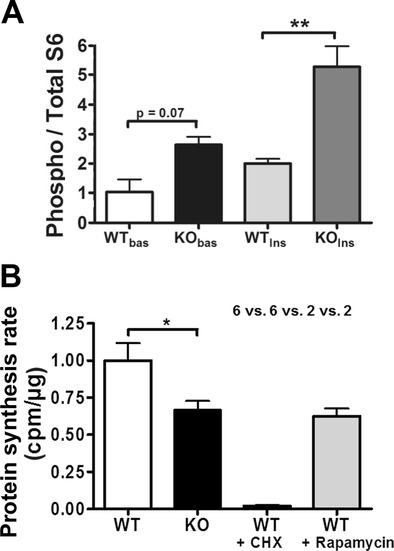
Ataxin-2 effects on global translation regulation and activity. **a** The ratio of ribosomal S6 phosphorylation normalized to total S6 content increased in *Atxn2* ^−/−^ cells. MEF cells from WT or KO animals were serum-starved for 24 h, and S6 phosphorylation was measured at basal condition (bas) or after incubation with insulin (Ins) over 10 min. A trend towards increased S6 phosphorylation was observed in KO MEFs at basal condition after starvation, and a highly significant increase of S6 phosphorylation was measured after insulin treatment (*n* = 2–3 MEF lines per genotype). **b** ATXN2 deficiency reduced basal mRNA translational activity. The incorporation of [S35]-labelled methionine/cysteine was quantified in MEFs to assess global protein synthesis rates. Cycloheximide (CHX) or rapamycin were applied 90 min prior to labelling to some cell lines. KO MEFs showed a reduction in protein synthesis by 34 % in comparison to WT MEFs (*n* = 6 MEF lines per genotype in 3 independent technical replicates). As control and comparison, the translation elongation inhibitor CHX diminished translation to 2 %, the mTOR pathway inhibitor rapamycin to 63 % (*n* = 2 MEF lines)

In order to understand whether these mRNA changes enhance ribosomal translation activity or are part of a compensatory effort to maintain translation homeostasis and to elucidate at which step within this pathway the ATXN2 deficiency might lead to a block, translation control and global protein synthesis were investigated in cells from 3 *Atxn2* ^*−/−*^ versus 3 *Atxn2* ^+/+^ mice. Because public databases on oligonucleotide microarrays and on serial analysis of gene expression (SAGE) document moderate *Atxn2* expression in skin and given that our preliminary experiments confirmed substantial *Atxn2* expression in skin fibroblasts [25], we employed mouse embryonal fibroblasts (MEFs). The experiments focused on ribosomal S6 protein as key modulator of translation initiation, which shows perfect co-sedimentation with ATXN2 in the brain [26]. As a first cellular phenotype, the translation control was investigated in KO versus WT MEFs by assessing the phosphorylation of S6 after 24-h serum deprivation (basal state) and after 10-min incubation with insulin (maximal state). A trend to increased phospho-S6 levels was observed at basal state (2.56-fold, *p* = 0.072), the increase becoming significant after insulin treatment (2.63-fold, *p* = 0.0097) (Fig. 4a). As the S6 kinase activity controls the ribosomal biogenesis transcriptional program [60], these experimental data are in agreement with the previous expression effects, jointly suggesting that the KO cells make enhanced efforts for mRNA translation.

S6 phosphorylation has been reported to modulate assembly of the translation initiation complex [61], while S6 deletion was found to influence cell cycle progression after mitogen stimulation more than cell size growth [62, 63]. As a second cellular phenotype therefore, general protein synthesis was investigated through incorporation rates of radioactively labelled amino acids into newly synthesized proteins of MEF cells. The absence of ATXN2 led to a moderate reduction of incorporation rates by 34 % (*p* = 0.029), similar to the effect of the mTOR kinase inhibitor drug rapamycin (reduction 37 %), while the translation elongation inhibitor drug cycloheximide (CHX) produced a complete suppression (by 98 %) (Fig. 4b). Thus, in spite of the elevated cellular ribosomal machinery and the enhanced translation signalling, the absence of ATXN2 caused a decrease in global protein synthesis rates.

## Discussion

We conducted an unbiased study to clarify, whether ATXN2 modulates specific RNAs or has global effects, and whether it acts as repressor or activator of translation. In view of the presence of ATXN2 as RNA-binding protein in stress granules, an ATXN2 role as a repressor of translation had been discussed by experts [64]. In contrast, recent experimental assessment of this postulate showed ATXN2 to act on the *PERIOD* transcript as an activator of translation rate [43, 44], and conversely, the depletion of ATXN2 was observed to reduce the stability and translation of AU-rich element containing mRNAs in vitro and in silico [59, 65]. Beyond such an effect on an individual mRNA, our data now consistently show global translation effects of ATXN2, as expected for a protein interactor of PABPC1. The absence of ATXN2 enhanced the abundance of the translation machinery and also the generic signals driving translation. In cells under growth stimulation, ATXN2 depletion potentiated the phosphorylation drive of translation, but in spite of this effort, the overall amino acid incorporation rates during protein synthesis were deficient. Thus, the formation of stress granules in times of cellular stress, with the sequestration of ATXN2/PABPC1/the small ribosomal subunit/mRNAs, may be interpreted as parallel efforts to reduce translational activity.

The formation of stress granules in dependence of ATXN2 appears to be highly conserved between man and yeast. In *S. cerevisiae*, the translational activator target of rapamycin complex 1 (TORC1) was found sequestrated together with the ATXN2 orthologue Pbp1 into stress granules [51]. Indeed, our murine data confirm that ATXN2 interacts with mammalian target of rapamycin (mTOR) signalling pathways, because ribosomal S6 phosphorylation is mTOR dependent [66] and is excessive in *Atxn2* ^−/−^ MEFs after insulin stimulation. Thus, yeast and mouse evidence jointly suggests that ATXN2 blunts mTOR signalling. It is also important to note that stress granule components can be recruited to lipid droplets. A study of hepatitis C virus (HCV) infection in a hepatocyte cell line showed the HCV production factory around lipid droplets to sequester P-body elements and the stress granule components ATXN2, PABPC1 and G3BP1 [67]. Thus, the pathways of mRNA quality control and decay are intertwined with the recruitment of lipid energy reserves during periods of cell stress. Therefore, our observation that ATXN2 depletion modifies the abundance of the lipid droplet regulators perilipin-3 (*Plin3*) and of the apolipoprotein secretion factor *Mttp*, of the RNA decay factor *Dcps* and of the tRNA splicing factor *Lsm12* appears meaningful in this context. These current findings were very similar to our previous observations in a project on the stress granule seeding factor TIA-1: the transcriptome profiling of mouse brain with genetic *Tia1*-ablation demonstrated from 1.6-fold until 3.5-fold alterations of the lipid droplet trafficking factor perilipin-4 (*Plin4*), the RNA decay factor *Dcp1b* and the tRNA splicing factor *Tsen2* [68]. The relevance of ATXN2 for the secretion of lipid trafficking factors was also shown in a recent study of the blood plasma proteome of SCA2 patients, where the significant dysregulation of 9 factors was documented, including a 4-fold decrease in apolipoprotein C3, a 3-fold decrease in apolipoprotein A1, a 2-fold decrease in aplipoprotein C2 and a > 4-fold increase in apolipoprotein E [69]. Ataxin-2 therefore appears to play a double role for mRNA translation and processing on the one hand, and for lipid recruitment and storage on the other hand, two crucial pathways during cell stress.

The mechanistic details, how the absence of ATXN2 exerts effects on translation and on the abundance of the ribosomal translation factors, remain unclear. ATXN2 could modulate the mRNA levels through direct interaction and decay modulation [59] or through its reported action as transcription factor in the nucleus [31]. But the upregulated abundance of translation factors could simply represent an indirect compensatory effort to overcome a blocked translation step. To our knowledge, only two transcription factors were previously reported to control in coordinated manner the transcript levels of ribosomal proteins and eukaryotic translation initiation/elongation factors in mammals in a coordinated manner. First, epidermal growth factor (EGF) stimulation via ERK phosphorylation of the transcription factor upstream binding factor (UBF) leads to an immediate activation of ribosomal transcription [70]. This can easily be linked to ATXN2, as we already showed the internalisation of activated EGF receptors and the levels of associated signalling molecules GRB2 and SRC to be modified by mutations of ATXN2 [24, 25]. Second, overexpression of the UBF co-activators c-Myc [71] or n-Myc within 4 h enhances the transcript levels of most ribosomal proteins [72]. This observation also ties in with our data about ATXN2 effects on ribosomal transcripts, because ATXN2 overexpression antagonizes n-Myc gene amplification, thus promoting the spontaneous regression of pediatric neuroblastoma tumors [73]. Both c-Myc translation and UBF levels are known to depend on mTOR signalling [74]. Thus, ATXN2 seems to act in concert with UBF, n-Myc and mTOR in modifying the transcriptional control of ribosomal machinery. In conclusion, a translation block due to ATXN2 depletion could trigger efforts to maintain protein synthesis within a range of homeostasis via these transcription factors and could explain the observations as indirect effects.

It remains unclear whether the changes of ribosomal translation and protein synthesis in ATXN2-ablated tissue underlie the previously reported neuroprotective effect of ATXN2 deficiency for motor neuron degeneration [4]. Indeed, recent screens in *S. cerevisiae*, *C. elegans* and *D. melanogaster* documented a role for ATXN2 as a generic modifier that affects multiple if not all neurodegenerative diseases, including several polyQ-triggered spinocerebellar ataxias [8]. It is therefore interesting to note that the dosage of ribosomes and translation factors was found to act as modifier of polyQ expansion protein aggregation, as recently shown in systematic *C. elegans* and *H. sapiens* studies [75]. Given that protein synthesis is a very energy-consuming process, its modulation may an important determinant of cell metabolism and atrophy. Thus, our novel data might be relevant for the neurodegenerative disease process and might provide neuroprotective insights.

While no data are available yet from patient tissues of the rare disease SCA2 regarding ribosomal translation, it is tempting to speculate that the SCA2-specific neurodegeneration pattern might be partially explained through the influence of ATXN2 mutations on global protein synthesis. The affected cerebellar Purkinje and brainstem olivo-pontine neurons, midbrain dopaminergic neurons, as well as spinal and cortical motor neurons are among the largest neurons of the nervous system, with characteristically large rER complexes (named Nissl substance or tigroid bodies) for ribosomal translation. These magnocellular neurons are the first to undergo cell death in SCA2, already at presymptomatic and initial stages of disease [76–79]. Thus, our findings are in agreement with known features of ATXN2-associated diseases, but experimental verification in human has to await the availability of frozen nervous tissue affected by this rare disorder.

Overall, our mouse mutant data show that the chronic depletion of ATXN2 impairs the rate of amino acid incorporation during mRNA translation for protein synthesis, while triggering responses in the liver and cerebellum to maintain global translation and bioenergetics through the transcriptional upregulation of specific factors in the pathways of ribosomal biogenesis/translation initiation/ER secretion/lipid trafficking.

## Materials and methods

### Mouse breeding and dissection

Animals were bred and aged in individually ventilated cages with continuous health monitoring, 4–6 animals per cage, under a 12-h light cycle with food (Ssniff M-Z, calories from protein 36 %, fat 11 % and carbohydrates 53 %) and water provided ad libitum. Mice were housed in accordance with the German Animal Welfare Act, the Council Directive of 24 November 1986 (86/609/EWG) with Annex II and the ETS123 (European Convention for the Protection of Vertebrate Animals) at the FELASA-certified Central Animal Facility (ZFE) of the Frankfurt University Medical School. All analyses were performed on male mice with a mixed C57BL/6_129/Ola background. Genotyping was performed with tail biopsies by PCR with three sets of primers as previously described [9].

### RNA preparation and cDNA synthesis

After cervical dislocation, the cerebellum and liver were dissected from homozygous wild-type (*Atxn2* ^+/+^) and knockout (*Atxn2* ^−/−^) mice at 6 and 24 weeks of age. Total RNA was extracted from these tissues by homogenization in 1 ml of Trizol® Reagent per 50–100 mg of tissue using a Pellet Pestle® Motor tissue homogenizer (Kontes, The Glass Company). One-microgram total RNA was digested with a DNase I Amplification Grade Kit (Invitrogen, Karlsruhe) in a reaction volume of 10 μl per tube in order to eliminate DNA during RNA purification prior to reverse transcription (RT-PCR) amplification. cDNA synthesis was performed with the Fermentas Life Sciences First Strand cDNA Synthesis Kit as instructed in the manual.

### Unbiased oligonucleotide microarray chip transcriptome profiling

The cerebellum and liver were dissected from 4 male homozygous wild-type (*Atxn2* ^+/+^) and 4 knockout (*Atxn2* ^−/−^) mice at 6 and 24 weeks of age. Double-stranded cDNA was synthesised from 1 μg of total RNA and was linearly amplified and biotinylated using the One-Cycle Target Labeling Kit (Affymetrix, Santa Clara, CA) according to the manufacturer’s instructions. Fifteen micrograms of labelled and fragmented cRNA was hybridized to MOE430 2.0 Gene Chip® oligonucleotide microarrays (Affymetrix) at MFTServices (Tübingen), thus detecting 39,000 transcripts and variants corresponding to 34,000 mouse genes. After hybridization, the arrays were washed and stained in a Fluidics Station 450 (Affymetrix) with the recommended washing procedure. Biotinylated cRNA bound to target molecules was detected with streptavidin-coupled phycoerithrin, biotinylated anti-streptavidin IgG antibodies and again streptavidin-coupled phycoerithrin according to the protocol. Arrays were scanned using the GCS3000 Gene Chip scanner (Affymetrix) and GCOS 1.4 software. Scanned images were subjected to visual inspection to control for hybridisation artifacts and proper grid alignment and analysed with Genespring (Agilent Technologies) and Expression Console MAS5, Microarray Suite 5.0 (Affymetrix), to generate report files for quality control. To define the influence of the factor genotype or age on the transcriptome, linear models were applied [80, 81]. For the mathematical-statistical assessment of data, their visualisation and functional correlation, the software platform R (version 2.5.0) and the pertinent bioconductor packages Affy, Just.RMA, and Limma (www.bioconductor.org) and diverse tools at the panther website (http://www.pantherdb.org) were used. Initially, the expression data from all chips were normalized with the RMA (Robust Microchip Average) to yield log2-transformed signal values. Global gene expression was compared between chips using scatter plots and Pearson’s R correlation coefficients. The signal values were then averaged for the individual subgroups, and differences in expression level were calculated in log2 space (M-values). Differences between subgroups were extracted as contrasts and analysed with the moderated F-test (empirical Bayes method) including a correction step for multiple testing with the 5 % FDR-based method of Benjamini and Hochberg [82]. To attribute significant effects to individual genes, a decision matrix was generated based on the function decide tests within the Limma option nestedF, where significant upregulations or downregulations are represented by values of 1 or −1, respectively.

### Validation of quantitative real-time reverse transcriptase polymerase chain reaction

Quantitative real-time reverse transcriptase polymerase chain reaction (qPCR) was performed using a GeneAmp® 5700 Sequence Detection System (Applied Biosystems, CA USA) with 96-well Optical Reaction Plates (Applied Biosystems, CA USA). Twenty-microliter final reaction volume per well contained 25–30 ng cDNA, TaqMan® Universal PCR Master Mix, No AmpErase® UNG and primers and probes in pre-designed TaqMan® Gene Expression Assays. The following assays were used: *Pabpc1*, Mm00849569_s1; *Nop10*, Mm00777618_g1; *Rps10*, Mm02391992_g1; *Rps18*, Mm02601777_g1; *Rpl14*, Mm00782569_s1; *Rpl18*, Mm01197265_g1; *Gnb2l1*, Mm01291968_g1 and Mm01291084_m1; *Eif2s2*, Mm00782672_s1; *Eif3e*, Mm01700222_g1; *Eif3s6*, Mm01700222_g1; *Eif4b*, Mm00778003_s1; *Sec61b*, Mm00834975_g1; *Srp14*, Mm00726104_s1; *Ssr1*, Mm00503135_m1. All assays were run in triplicate. *Tbp* (Mm 00446973_m1) was used as an endogenous control in all experiments and was run in wells separate from the target gene assays. The PCR conditions were 50 °C for 2 min and 95 °C for 10 min followed by 40 cycles at 95 °C for 15 s and 60 °C for 40 s. Analysis of relative gene expression data was performed using the ΔΔCT method.

### Quantitative immunoblot analysis

Liver tissue from 4 wild-type and 4 homozygous *Atxn2* knockout mice was weighed and processed. It was homogenized with a motor pestle in 10 vol. RIPA buffer [50 mM Tris–HCl (pH 8.0), 150 mM NaCl, 1 mM EDTA, 1 mM EGTA, 1 % Igepal CA-630 (Sigma), 0.5 % sodium deoxycholate, 0.1 % SDS, 1 mM PMSF, Complete Protease Inhibitor Cocktail (Roche)] and incubated on ice for 15 min. After centrifugation at 4 °C and 16,000×*g* for 20 min, the supernatant was stored (RIPA-soluble fraction), and the remaining pellet was dissolved in ½ vol. 2× SDS buffer [137 mM Tris–HCl (pH 6.8), 4 % SDS, 20 % glycerol, Complete Protease Inhibitor Cocktail (Roche)] by sonification followed by 10 min of centrifugation at 16,000×*g*. The resulting supernatant was stored as RIPA-insoluble fraction.

The protein concentration of the samples was measured using the standard Bradford protein assay [83]. Bovine serum albumin (BSA) dilutions were used as standards to construct the calibration curve. Prior to every gel electrophoresis experiment, 2× loading buffer was added to the tissue homogenates, which were then heated at 95 °C for 5 min.

The samples were analysed by sodium dodecylsulfate polyacrylamide gel electrophoresis (SDS-PAGE) according to Laemmli, loading 10 μg protein per lane [84]. The time required for transfer of the protein pattern to the PVDF membrane at 100 V varied between half an hour for small proteins <20 kDa and an hour for larger proteins. After electroblotting, unspecific binding sites on the PVDF membrane were blocked for 1 h at room temperature in a solution of 5 % dry milk powder in PBS containing 0.05 % Tween 20 (PBS/T) and then incubated over night at 4 °C with the following antibodies diluted in a mixture of 2.5 ml PBS/T and 2.5 ml of 5 % dry milk powder in PBS/T: mouse monoclonal anti-Ataxin-2 (1:500, BD Biosciences), rabbit polyclonal anti-Phospho-eIF4B (1:1000, Cell Signaling), rabbit polyclonal anti-PABP (1:1000, Abcam UK), rabbit monoclonal anti-NOP10 (1:1000, LSBio), rabbit polyclonal anti-RPL8 (1:2000, GeneTex), mouse polyclonal anti-RPL18 (1:500, Abnova, Taiwan Corporation), rabbit polyclonal ribosomal protein anti-L26 (1:1000, Cell Signaling), rabbit polyclonal ribosomal protein anti-S3 (1:1000, Cell Signaling), rabbit polyclonal ribosomal protein anti-S6 (1:1000, Cell Signaling), rabbit polyclonal ribosomal protein anti-S10 (1:2000, ThermoFisher), rabbit polyclonal anti-RPS18 (1:1000, Acris), rabbit polyclonal anti-RACK1 (1:1000, Cell Signaling), mouse monoclonal anti-RACK1 (1:2500, BD Transduction), rabbit polyclonal anti-EIF2S2 (1:2000, proteintech), rabbit polyclonal anti-SRP14 (1:1000, ProteinTech Group, Inc.), rabbit polyclonal anti-SRP14 (1:300, Assay Designs), mouse monoclonal anti-SSR1 (1:500, Novus Biologicals) antibody and mouse monoclonal anti-beta-ACTIN (1: 10000, Sigma) as a loading control. Following incubation with the primary antibody, the membranes were washed (3 × 10 min in PBS/T) and then incubated with the secondary antibodies conjugated to horse radish peroxidase (HRP) for 1 h at room temperature (anti-mouse-IgG-horseradish peroxidase (1:10,000, GE Healthcare), anti-rabbit-IgG-horseradish peroxidase (1:10,000, Amersham Biosciences), donkey-anti-goat-IgG-horseradish peroxidase (1:30,000, Santa Cruz Biotechnology, Inc.). After binding of the secondary antibody, the blots were washed again in PBS/T (3 × 10 min), and ECL detection (Supersignal West Pico Chemiluminescent Substrate, Pierce) was performed at room temperature according to the manufacturer’s protocol, with varying exposure times to avoid film sensitivity or saturation problems as well as non-linear effects. The images were digitalized on a scanner (Epson) and densitometry performed with the proprietary ImageMaster Total Lab 2.00 software (AmershamPharmacia). After normalization of candidate protein values versus beta-actin values from the identical membrane in EXCEL, the changes were evaluated in GraphPad statistics and plotting.

### Ribosomal S6 phosphorylation

Mouse embryonal fibroblasts (MEFs) were generated as previously described [24]. To investigate the ribosomal S6 phosphorylation status in MEFs, confluent cells were serum-starved for 24 h, and either left untreated or incubated with 100 nM insulin for 10 min. The phosphorylation status of S6 was measured with the PathScan Phospho-S6 Ribosomal Protein (Ser235/236) Sandwich ELISA Kit (#7205, Cell Signaling, Danvers, USA) according to manufacturer’s protocol. One micromolar PMSF was added to the cell lysis buffer to prevent protein degradation.

### Global protein synthesis after deprivation and stimulation in vitro

For the analysis of global protein synthesis rates, 2 × 10^5^ MEFs were seeded on 6-well plates the day before the experiment. Cells were then deprived of methionine and cysteine for 30 min and labelled by addition of 20 μCi/ml [^35^S] EasyTag Express Protein Labeling Mix for 40 min (Perkin Elmer, Waltham, MA, USA). For the treatment with inhibitors, cells were supplemented 30 min before the labelling with 1 μM cycloheximide (CHX, Sigma-Aldrich, Munich, Germany) or 20 nM rapamycin (LC Laboratories, Woburn, MA, USA). After washing twice with ice-cold PBS, cells were lysed in RIPA and precipitated with ice-cold 10 % TCA on GF/C filters (Whatman, Dassel, Germany). After washing twice with ice-cold 5 % TCA and once with methanol, filters were dried and subjected to liquid scintillation counting (Perkin-Elmer, Boston, USA).

### Statistical analysis

The Graph-Pad software package (version 4.03, GraphPad Software Inc., San Diego, California, USA) was used to perform the non-parametric Mann-Whitney test or the parametric Student’s *t* test to represent data with bar graphs and to illustrate mean values and standard deviations. Significant differences were highlighted with asterisks (*p < 0.05; **p < 0.01; ***p < 0.005).

## Electronic supplementary material

Below is the link to the electronic supplementary material.

Supplemental Table 1 Transcriptome profiling by oligonucleotide microarray chips.
Significant upregulations of non-anonymous genes with consistency between liver and cerebellum at ages 6 and 24 weeks are shown (each of 4 KO versus 4 WT), ordered first by pathways and then alphabetically. The columns from the left represent the pathways, the oligonucleotide ID on Affymetrix chips, the Gene Name, the Gene Symbol (bold letters for ATXN2 interactor/competitor proteins), the results from F-test statistics with F-values, nominal P-values, adjusted P-values (after correction for multiple testing, with grey highlighting of particularly significant results beyond E-10), the M-values (log2 fold changes) for 6-week-old liver (6L) and cerebellum (6C) as well as 24-week-old liver (24L) and cerebellum (24C) (highlighting in grey for changes >2-fold, i.e. log2 >1), followed by a value for the average expression level observed for each gene, and finally functional comments by the authors. (XLSX 26 kb)
